# TIE1 Promotes Primary Tumor Growth by Inhibiting Apoptosis and Activating the AKT‐p70S6K Signaling Pathway in Breast Cancer

**DOI:** 10.1111/gtc.70062

**Published:** 2025-11-14

**Authors:** Kazushi Azuma, Takaya Matsuyama, Shinya Watanabe, Kentaro Semba, Jun Nakayama

**Affiliations:** ^1^ Department of Life Science and Medical Bioscience School of Advanced Science and Engineering, Waseda University Tokyo Japan; ^2^ Translational Research Center, Fukushima Medical University Fukushima Japan; ^3^ Department of Oncogenesis and Growth Regulation Research Institute, Osaka International Cancer Institute Osaka Japan

**Keywords:** AKT‐p70S6K pathway, breast cancer, orthotopic xenograft, TIE1

## Abstract

Triple‐negative breast cancer (TNBC) is the most aggressive molecular subtype among all breast cancer types. Its treatment remains a significant challenge due to the lack of clearly defined molecular targets. We previously reported that lung‐metastatic cell lines, established via orthotopic transplantation of a TNBC cell line, showed high expression of the TIE1 receptor‐tyrosine kinase. In this study, we demonstrated that TIE1 expression correlates with poor prognosis in breast cancer patients and is highly elevated in the Claudin‐low subtype, which largely overlaps with TNBC. Notably, TIE1 expression promoted tumorigenicity in a breast cancer cell line. Furthermore, in primary tumors formed by TIE1‐expressing cells, we observed TIE1 cleavage, reduced apoptosis, and activation of the AKT‐p70S6K signaling pathway. Our findings suggest that TIE1 may serve as a potential molecular target and biomarker for Claudin‐low type breast cancer, and further research could have significant implications for its treatment.

## Graphical abstract

Highly lung‐metastatic breast‐cancer cells with elevated TIE1 expression exhibit increased tumorigenicity. In primary tumors, TIE1 undergoes cleavage and promotes cancer cell survival by activating the AKT–p70S6K signaling pathway.

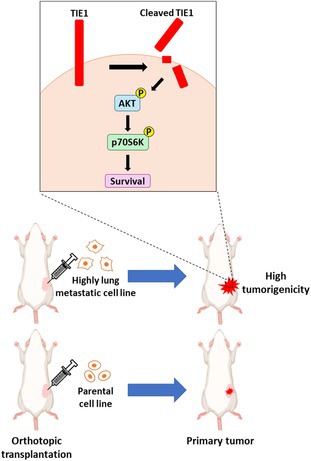

## 1 Introduction

Breast cancer is traditionally classified into three subtypes based on the expression levels of biomarkers, including hormone receptors (HR), human epidermal growth factor receptor 2 (HER2), and Ki67. These subtypes include HR‐positive breast cancer, HER2‐positive breast cancer, and triple‐negative breast cancer (TNBC) (Sarhangi et al. 2022). Subsequently, more detailed classification methods have been developed. Among them, genomic profiling divides breast cancer into six molecular subtypes—Luminal A, Luminal B, HER2‐enriched, Basal‐like, Claudin‐low, and normal‐like—based on gene expression patterns (Voutsadakis 2023). Subtype classification is closely linked to treatment strategies and prognosis (Harbeck and Gnant 2017). Although hormone therapy and molecular‐targeted treatments have significantly improved outcomes in breast cancer, they are largely ineffective for TNBC (Dent et al. 2007; Lin et al. 2008; Ben‐Dror et al. 2022). In recent years, neoadjuvant chemotherapy (NAC) has been applied to some TNBC patients, and PARP inhibitors have emerged as a potential treatment option (Mahtani et al. 2021). While NAC is considered a standard approach for early‐stage TNBC, the pathological complete response rate is only around 30%–40%, and a considerable proportion of TNBC patients do not respond effectively (Cortazar et al. 2014; Oshi et al. 2020). Additionally, PARP inhibitors show particular efficacy in TNBC patients with BRCA mutations, which account for approximately 15% of TNBC cases (Hartman et al. 2012; Engel et al. 2018; McCann and Hurvitz 2018). Given these limitations, identifying new therapeutic targets for TNBC remains an urgent priority.

We previously established highly lung‐metastatic breast‐cancer cell lines (OX‐LM cell lines) derived from orthotopic xenografts (OX) of MDA‐MB‐231 (MM231), a representative triple‐negative and Claudin‐low subtype breast‐cancer cell line (Nakayama et al. 2017). Among them, LM05 exhibited greater tumorigenicity compared to MM231 and lung‐metastatic cell lines established by tail vein injection (TV‐LM cell lines) (Hayashi et al. 2023). Comparative analysis between OX‐LM and TV‐LM cell lines revealed that tyrosine kinase with immunoglobulin‐like and EGF‐like domains 1 (TIE1) was highly expressed in OX‐LM cell lines. TIE1 is a receptor‐tyrosine kinase (RTK) belonging to the TIE family, primarily expressed in endothelial cells (Partanen et al. 1992). Its primary function is to maintain vascular stability by regulating the activation of TIE2, another member of the TIE family (Korhonen et al. 2016; Wang et al. 2022; Cao et al. 2023). One of the regulatory mechanisms of TIE1 is proteolytic cleavage of its ectodomain. Extracellular domain shedding of TIE1 is primarily mediated by metalloproteases, including members of the ADAM and MMP families. While direct evidence supports the involvement of the ADAM family, the precise mechanisms remain to be fully elucidated (Hayashida et al. 2010; Huang 2021; Du et al. 2024). In cancer research, TIE1 has been found to be upregulated in various cancers, including breast cancer (Lin et al. 1999; Rees et al. 2007; Torigata et al. 2017; Wei et al. 2024). Moreover, elevated plasma TIE1 levels have been associated with poor prognosis in metastatic breast cancer (Tiainen et al. 2019). In ovarian cancer, TIE1 expression promotes cell proliferation and survival through the phosphatidylinositol 3‐kinase (PI3K)–AKT signaling pathway (Zhang et al. 2020), and contributes to cisplatin resistance by enhancing nucleotide excision repair through the expression of xeroderma pigmentosum complementation group C (XPC) regulated by the transcription factor KLF5 (Ishibashi et al. 2018). However, the functional role of TIE1 in breast cancer has not yet been extensively investigated.

In this study, we investigated the function of TIE1 using MM231 and OX‐LM cell lines and revealed its contribution to tumorigenicity. We further identified a signaling pathway whose activation correlates with primary tumor growth and proposed a model in which proteolytic cleavage of TIE1 activates this pathway.

## 2 Results

### 2.1 OX‐LM Cell Lines Are the Aggressive Model of Claudin‐Low Type Breast Cancer With TIE1 Expression

**FIGURE 1:**
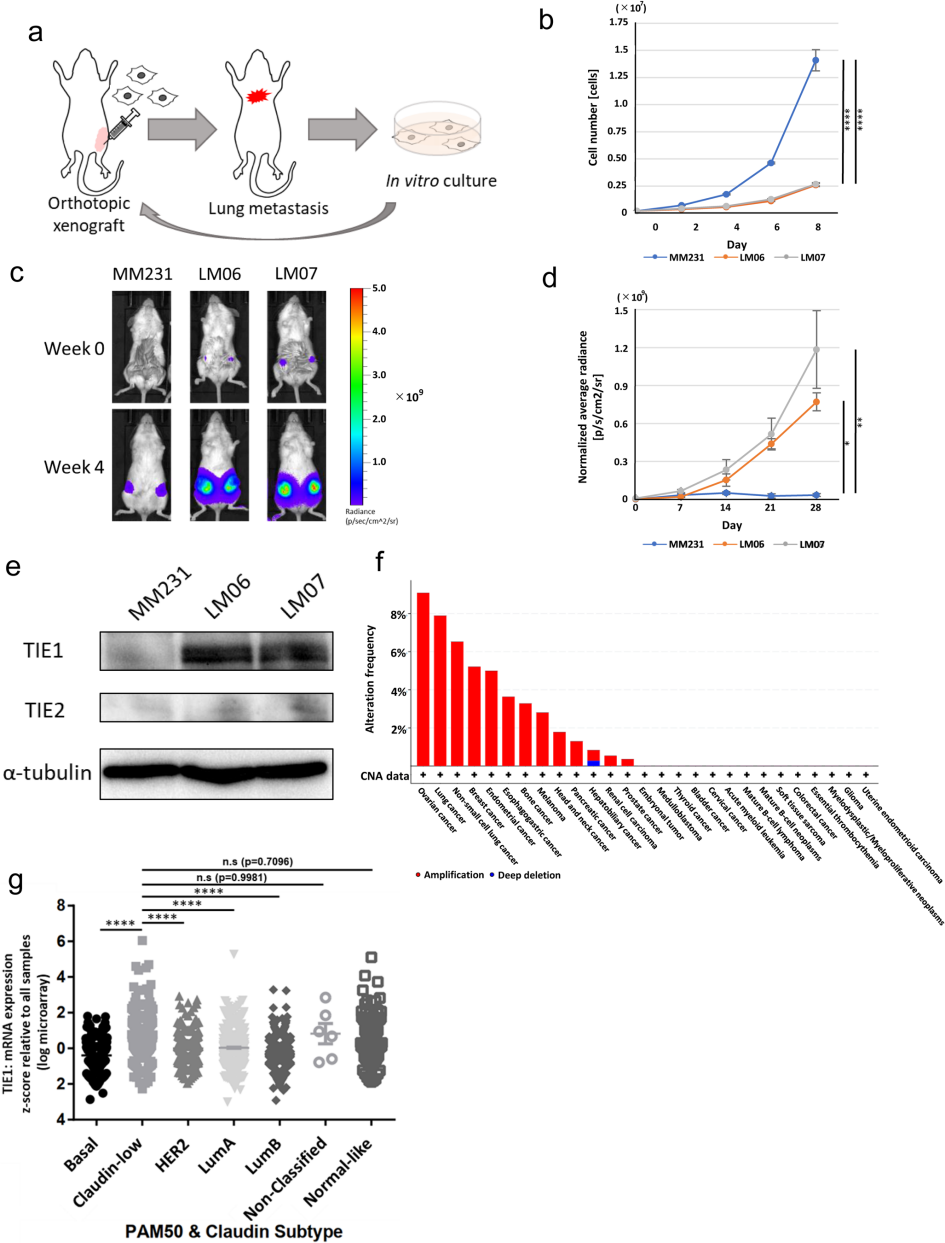
TIE1 is highly expressed in OX‐LM cell lines with high tumorigenicity and in Claudin‐low type breast cancer. (a) Schematic of the method used to establish highly lung‐metastatic cell lines via OX. (b) Cell growth curves of MM231, LM06, and LM07 cell lines (*n* = 3). One‐way ANOVA followed by Holm–Sidak's multiple comparisons test. (c) Representative in vivo luminescence images of primary tumors in OX models of MM231‐Venus, LM06‐shGFP, and LM07‐shGFP cell lines (injection site; MM231‐Venus: *n* = 6, LM06‐shGFP: *n* = 4, LM07‐shGFP: *n* = 6). (d) Tumor growth curves with intensity of bioluminescence (MM231‐Venus: *n* = 6, LM06‐shGFP: *n* = 4, LM07‐shGFP: *n* = 6). One‐way ANOVA followed by Holm–Sidak's multiple comparisons test. (e) Western blot analysis of TIE1 and TIE2 in MM231, LM06, and LM07 cell lines. (f) Copy number alteration analysis of TIE1 across pan‐cancer clinical datasets from TCGA and ICGC (total: *n* = 2703, ovarian cancer: *n* = 110; lung cancer: *n* = 38; non‐small cell lung cancer: *n* = 46; breast cancer: *n* = 211; endometrial cancer: *n* = 20; esophagogastric cancer: *n* = 165; bone cancer: *n* = 61; melanoma: *n* = 107; head and neck cancer: *n* = 56; pancreatic cancer: *n* = 309; hepatobiliary cancer: *n* = 358; renal cell carcinoma: *n* = 186; prostate cancer: *n* = 275; embryonal tumor: *n* = 120; medulloblastoma: *n* = 21; thyroid cancer: *n* = 48; bladder cancer: *n* = 23; cervical cancer: *n* = 20; acute myeloid leukemia: *n* = 16; mature B‐cell lymphoma: *n* = 103; mature B‐cell neoplasms: *n* = 93; soft tissue sarcoma: *n* = 34; colorectal cancer: *n* = 52; essential thrombocythemia: *n* = 27; myelodysplastic/myeloproliferative neoplasms: *n* = 26; glioma: *n* = 146; uterine endometrioid carcinoma: *n* = 24). (g) Comparative expression analysis of TIE1 among breast‐cancer subtypes in the METABRIC dataset: Basal (*n* = 209), Claudin‐low (*n* = 218), HER2‐positive (*n* = 224), luminal A (*n* = 700), luminal B (*n* = 475), normal‐like (*n* = 148), and non‐classified (*n* = 6). One‐way ANOVA followed by Tukey's multiple comparisons test. All data are presented as mean ± SEM. n.s., not significant. *p < 0.05, **p < 0.01, ****p < 0.0001.

Previously, we established three OX‐LM cell lines—LM05, LM06, and LM07—from the MM231 cell line (Nakayama et al. 2017). These lines were derived through two cycles of recovery from lung‐metastatic lesions formed by OX (Figure 1a). In vitro, the OX‐LM cell lines proliferated more slowly than MM231 (Figure 1b). To assess tumorigenicity, we compared MM231 with the OX‐LM cell lines, and found that the OX‐LM cell lines exhibited significantly higher tumorigenicity (Figure 1c,d; Figure S1a–c). These findings suggest that OX‐LM cell lines possess a heightened proliferative capacity specifically within the in vivo environment. In our previous study, TIE1 was shown to be highly expressed at the transcriptional level in OX‐LM cell lines (Nakayama et al. 2017). In this study, we found that TIE1 protein levels are consistently higher in OX‐LM cell lines than in MM231. (Figure 1e). To explore clinical relevance, we analyzed data from the Pan‐International Cancer Genome Consortium (ICGC)/The Cancer Genome Atlas (TCGA). Among various cancer types, breast cancer was the fourth most common to exhibit TIE1 amplification (Figure 1f). Furthermore, TIE1 was highly expressed in Claudin‐low breast cancers, the same subtype as MM231 (Figure 1g). From the above, OX‐LM cell lines represent a valuable model of Claudin‐low type TNBC characterized by TIE1 expression.

### 2.2 TIE1 Contribute Breast‐Cancer Malignancy by Promoting Primary Tumor Growth

**FIGURE 2:**
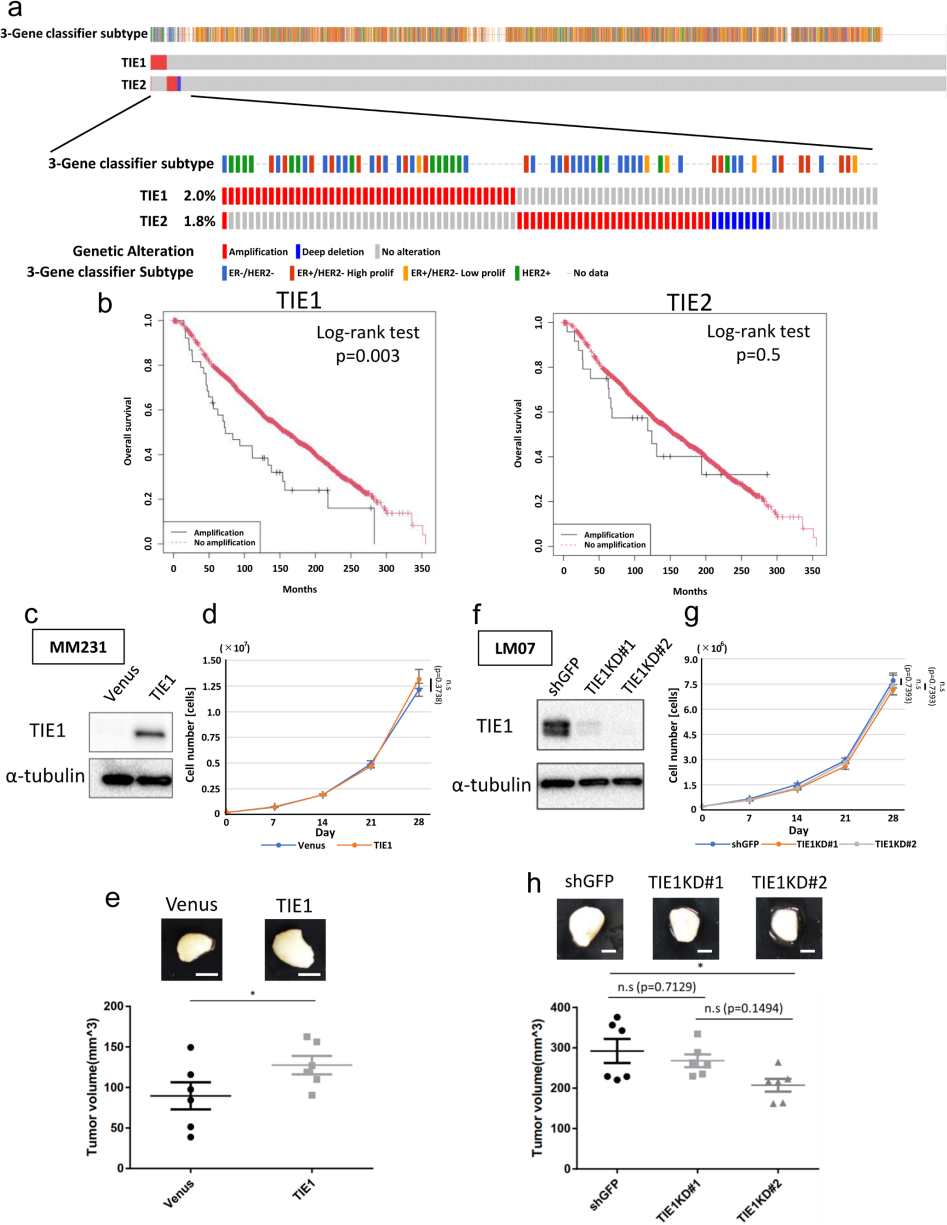
TIE1 amplification correlates with poor prognosis in breast cancer and promotes primary tumor growth. (a) OncoPrint showing the genomic status of TIE1 and TIE2 in the METABRIC dataset (total patient: *n* = 2173). (b) Survival analysis of METABRIC cohorts with or without TIE1 (amplification: *n* = 38; no amplification: *n* = 1942) and TIE2 (amplification: *n* = 24; no amplification: *n* = 1956) amplification, gene displayed using Kaplan–Meier plots. Log‐rank test (TIE1, *p* = 0.003; TIE2, *p* = 0.5). (c) Western blot analysis of TIE1 expression in MM231‐Venus and MM231‐TIE1 cell lines. (d) Cell growth curves of MM231‐Venus and MM231‐TIE1 cell lines (*n* = 4). One‐way ANOVA followed by Welch's *t*‐test. (e) Representative images of primary tumors (upper panel) and quantification of tumor volumes (lower panel) in MM231‐Venus and MM231‐TIE1 cell lines 7 weeks after from transplantation (MM231‐Venus: *n* = 6; MM231‐TIE1: *n* = 6). Welch's *t*‐test. (f) Western blot analysis of TIE1 in LM07‐shGFP, LM07‐TIE1KD#1, and LM07‐TIE1KD#2 cell lines. (g) Cell growth curves of LM07‐shGFP, LM07‐TIE1KD#1, and LM07‐TIE1KD#2 (*n* = 4). One‐way ANOVA followed by Holm–Sidak's multiple comparisons test. (h) Representative images of primary tumors (upper panel) and quantification of tumor volumes (lower panel) in LM07‐shGFP, LM07‐TIE1KD#1, and LM07‐TIE1KD#2 cell lines 4 weeks after from transplantation (LM07‐shGFP: *n* = 6; LM07‐TIE1KD#1: *n* = 6; LM07‐TIE1KD#2: *n* = 6). One‐way ANOVA followed by Tukey's multiple comparison test. All data are presented as mean ± SEM. n.s., not significant; *p < 0.05.

To elucidate the clinical significance of TIE1 in breast cancer, we analyzed its genomic profile using the Molecular Taxonomy of Breast‐Cancer International Consortium (METABRIC) cohort. Copy number alterations of TIE1 and TIE2 were visualized as an OncoPrint using cBioPortal (Figure 2a). Both TIE1 and TIE2 were found to be amplified in breast‐cancer patients; however, only TIE1 amplification was significantly associated with poor prognosis (Figure 2b). These findings suggest that TIE1 contributes to poor clinical outcomes in breast cancer independently of TIE2. To investigate the function of TIE1 in breast cancer, we established a TIE1‐overexpressing MM231 cell line (MM231‐TIE1) (Figure 2c). MM231‐TIE1 cells were orthotopically transplanted into NOD.CB‐17‐Prkdc‐scid/J (NOD‐SCID) mice to evaluate tumorigenicity. Although TIE1 expression in MM231 did not affect cell growth in vitro, it enhanced tumorigenicity in vivo (Figure 2d,e). Next, we performed TIE1 knockdown (KD) in LM07 cells and orthotopically transplanted these KD cells (Figure 2f). While TIE1 knockdown did not alter in vitro growth (Figure 2g), it significantly suppressed tumor growth in vivo (Figure 2h). However, it is noteworthy that TIE1 expression did not correlate with lung metastasis formation in LM07 (Figure S1d). Together, these results indicate that TIE1 promotes primary tumor growth but does not contribute to lung metastasis, suggesting its role in breast‐cancer progression is specific to primary tumor growth.

### 2.3 TIE1 Is Cleaved in Primary Tumor and Induce Activation of AKT‐p70S6K Signal Pathway

**FIGURE 3:**
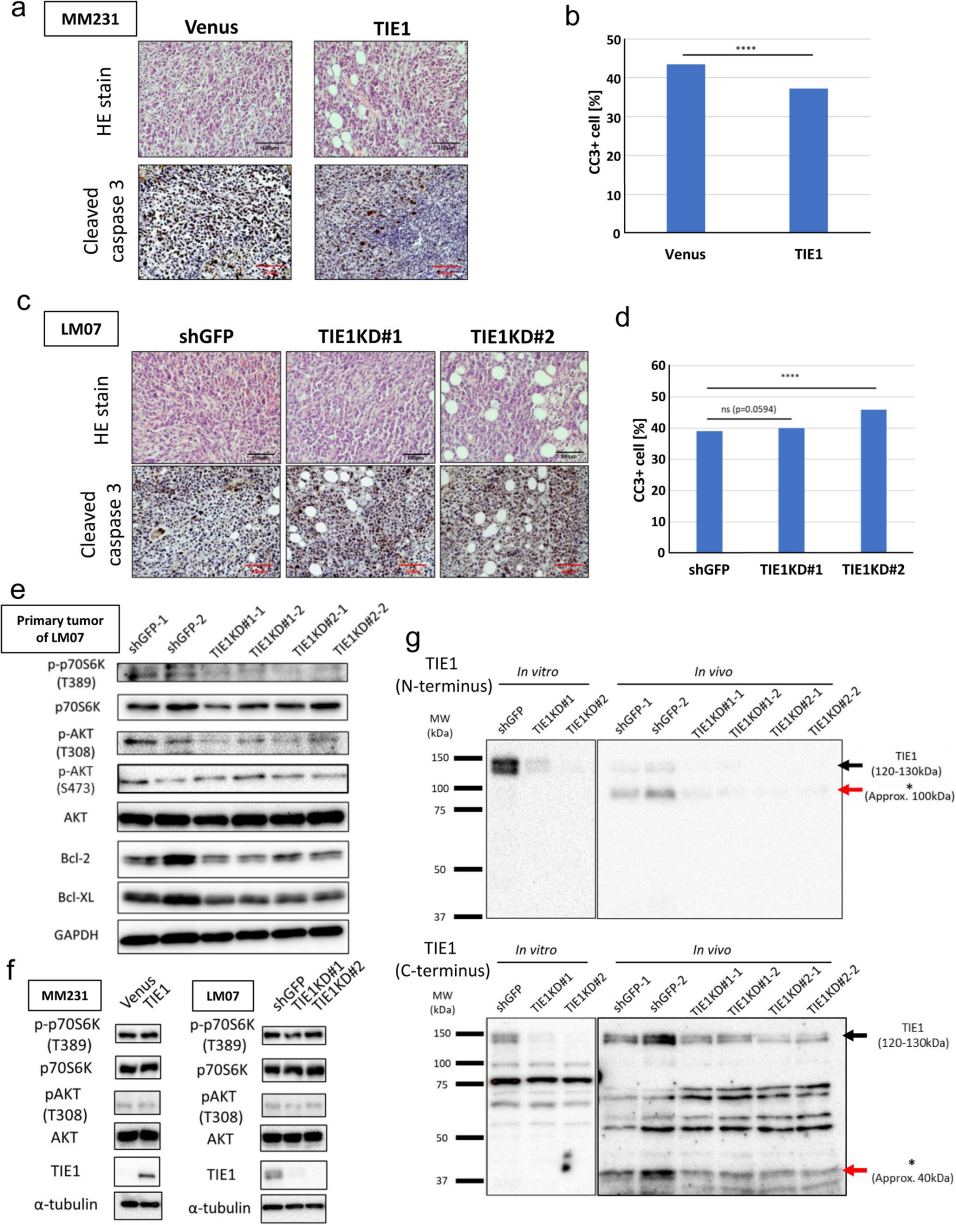
TIE1 suppresses apoptosis in breast‐cancer cells by activating the AKT–p70S6K signaling pathway. (a) Representative HE and IHC staining images of primary tumor tissues derived from orthotopically injected NOD‐SCID mice with MM231‐Venus and MM231‐TIE1 cell lines. (b) Quantification of CC3‐positive cells in primary tumors from orthotopically injected NOD‐SCID mice bearing MM231‐Venus or MM231‐TIE1 cells. The number of CC3‐positive cells was counted from three fields of view per tumor across *n* = 4 individual tumor sections and analyzed using the Chi‐squared test. (c) Representative HE and IHC staining images of primary tumor tissues derived from orthotopically injected NOD‐SCID mice with LM07‐shGFP, LM07‐TIE1KD#1, and LM07‐TIE1KD#2 cell lines. (d) Quantification of CC3‐positive cells in primary tumors from LM07‐shGFP, LM07‐TIE1KD#1, and LM07‐TIE1KD#2 groups. Counts were based on three fields of view per tumor from *n* = 3 individual sections and tested using the Chi‐squared test. n.s., not significant; ****p < 0.0001. (e) Western blot analysis of the AKT–p70S6K signaling pathway in primary tumors derived from LM07‐shGFP, LM07‐TIE1KD#1, and LM07‐TIE1KD#2 cell lines. Protein lysates were prepared from two tumors per cell line. (f) Western blot analysis of the AKT–p70S6K signaling pathway in MM231‐Venus and MM231‐TIE1 cells (Left panel) and in LM07‐shGFP, LM07‐TIE1KD#1, and LM07‐TIE1KD#2 cells (right panel). (g) Western blotting of TIE1 in primary tumors of LM07‐shGFP, LM07‐TIE1KD#1, and LM07‐TIE1KD#2 cell lines using two TIE1 antibodies: One recognizing the N‐terminus (upper panel) and the other recognizing the C‐terminus (lower panel). Red arrows indicate potential TIE1 fragments (single asterisk: N‐terminal fragment; double asterisk: C‐terminus fragment).

We performed immunohistochemistry (IHC) analysis on primary tumor tissues. To evaluate TIE1's role in apoptosis, we quantified cleaved caspase‐3 (CC3)‐positive cells (Figure S2a). The number of CC3‐positive cells was significantly reduced in MM231‐TIE1 tumors (Figure 3a,b; Figure S2b; Table S1). Consistently, CC3‐positive cells were significantly increased in LM07 following TIE1KD (Figure 3c,d; Figure S2c; Table S2). These findings suggest that TIE1 suppresses apoptosis in primary tumors. To explore this mechanism further, we obtained protein lysates from primary tumors of TIE1KD LM07 cell lines. To detect all protein signals, including secretory proteins, whole tumors were lysed and analyzed by western blotting. In TIE1KD tumors, the expression of anti‐apoptotic proteins Bcl‐2 and Bcl‐XL, as well as the phosphorylation levels of AKT (T308) and p70S6K, were reduced (Figure 3e). Interestingly, this pathway activity was not altered under in vitro culture conditions (Figure 3f). Previous studies have reported that TIE1 undergoes proteolytic cleavage in endothelial cells, and such cleavage often plays a role in RTK activation (Yabkowitz et al. 1997; Marron et al. 2007; Du et al. 2024). Based on this, we hypothesized that TIE1 might be activated via proteolytic cleavage. To test this, we performed Western blot analysis using lysates from in vitro cultured cells and in vivo tumors, employing two TIE1 antibodies: one targeting the N‐terminus and the other targeting the C‐terminus. As a result, we detected additional bands (indicated by red arrows) distinct from the full‐length TIE1 protein (black arrows) (Figure 3g). The intensity of these additional bands corresponded to the TIE1 expression levels in each cell line. Collectively, these results support the hypothesis that TIE1 is cleaved in vivo, leading to activation of the AKT–p70S6K signaling pathway and contributing to enhanced cell survival in primary breast tumors.

## 3 Discussion

Our findings propose a novel function for TIE1 in breast cancer, distinct from its established role in endothelial cells. First, we found that TIE1 promotes tumorigenicity in breast cancer. However, the tumorigenicity is unlikely to be driven by TIE1 alone. Although TIE1KD in OX‐LM cell lines reduced tumorigenicity, these cells still exhibited higher tumorigenicity than the parental MM231 line. In our previous study, NF‐κB‐inducing kinase (NIK) was also implicated in the tumorigenicity of LM05 cells (Hayashi et al. 2023), suggesting that the tumorigenicity of OX‐LM cell lines cannot be attributed to a single gene. Second, TIE1 may activate AKT‐mediated signaling independently of TIE2. We observed that TIE2 expression was not upregulated in OX‐LM cell lines (Figure 1e). Nevertheless, modulating TIE1 levels affected the tumorigenic capacity of breast‐cancer cells. Since heterodimerization between TIE1 and TIE2 inhibits TIE2 activation (Song et al. 2012), it is unlikely that TIE1 activates downstream signaling in OX‐LM cell lines through TIE2. Supporting this, previous studies have shown that TIE1 can independently activate the PI3K–AKT signaling pathway (Kontos et al. 2002; Zhang et al. 2020). Additionally, only TIE1 amplification—not TIE2—is associated with poor prognosis in breast‐cancer patients (Figure 2b), reinforcing the idea that TIE1 may contribute to tumor progression in a TIE2‐independent manner. Finally, our data suggest that TIE1 cleavage may trigger signaling activation. In lymphatic endothelial cells, the ectodomain (ECD) of TIE1 is cleaved by a disintegrin and metalloproteinase 17 (ADAM17) (Du et al. 2024). This initial cleavage is followed by intramembrane proteolysis via γ‐secretase, resulting in the release of a ~100 kDa ECD and generation of a ~42 kDa intracellular domain (Yabkowitz et al. 1997; Marron et al. 2007). Notably, a ~45 kDa c‐terminal fragment of TIE1 has been reported in MCF7 breast‐cancer cells (Rees et al. 2007). These are closely matching the molecular weight of the fragments we detected. Our result might provide a mechanism for TIE1 activation which is unknown so far.

We also demonstrated activation of the AKT–p70S6K signaling pathway. This pathway is known to be associated with cancer cell survival across multiple cancer types, including breast cancer (Zhu et al. 2014, 2018; Zhang et al. 2018). AKT promotes cell survival by phosphorylating and inhibiting pro‐apoptotic proteins such as GSK3, and by activating cyclic adenosine monophosphate (cAMP) response element‐binding protein (CREB), which regulates the expression of anti‐apoptotic genes such as Bcl‐2 and Bcl‐XL (Wang et al. 2019; Zarneshan et al. 2022). Our findings align with these previous reports and highlight the significance of TIE1‐mediated regulation of the PI3K–AKT signaling axis.

In this study, we showed that TIE1 expression promotes primary tumor growth primarily through the suppression of apoptosis. Moreover, we identified the AKT–p70S6K pathway as a downstream signaling cascade that correlates with TIE1 expression and cleavage in breast‐cancer cells (Figure 3e–g). Considering that Claudin‐low tumors account for approximately 30% of TNBC cases (Prat and Perou 2011), our findings suggest that TIE1 may represent a potential therapeutic target for a subset of TNBC patients, and that OX‐LM cell lines provide a valuable model of this Claudin‐low subtype characterized by TIE1 expression. While further investigations are needed to clarify the mechanisms of TIE1 cleavage and its clinical implications, our study suggests that TIE1 and its downstream signaling components hold promise as potential biomarkers and therapeutic targets in Claudin‐low type TNBC.

## 4 Materials and Methods

### 4.1 Cell Culture

MM231‐*mSlc7a1*‐luc2 (hereafter referred to as MM231), LM06, and LM07 cell lines were established as previously described (Nakayama et al. 2017). These cells were cultured in Roswell Park Memorial Institute 1640 medium (RPMI1640; Fujifilm Wako), supplemented with 10% fetal bovine serum (FBS; NICHIREI BIOSCIENCES INC.), 100 U/mL penicillin G (Meiji‐Seika Pharma Co. Ltd.), and 100 μg/mL streptomycin (Meiji‐Seika Pharma Co. Ltd.) at 37°C in a humidified atmosphere containing 5% CO_2_. Plat‐E cells, used for retroviral packaging and kindly provided by T. Kitamura (Institute of Medical Science, University of Tokyo), were maintained in Dulbecco's Modified Eagle Medium (DMEM) with 10% FBS, 100 U/mL penicillin G, and 100 μg/mL streptomycin under the same incubation conditions.

### 4.2 Western Blotting

To acquire cellular protein lysate, cells were lysed with SDS sampling buffer (3.5% SDS, 12% glycerol, 0.05 M Tris–HCl (pH 6.8), 0.003% bromo phenol blue, and 5% 2‐mercaptoethanol). To collect tumor protein lysate, tumors were physically homogenized with RIPA buffer (0.1% SDS, 1% NP‐40, 1 mM EDTA, 150 mM NaCl, 0.5% sodium deoxycholate, 2.5 mM Tris–HCl (pH 7.4), 1 mM PMSF, 1.5 mM Na_3_VO_4_, 10 mM NaF). Then, tumor homogenized solutions were rotated at 4°C for 30 min, and sonicated for 3 min. Finally, the supernatant of tumor homogenized solutions was diluted to 10 μg/15 μL protein concentration. Western blotting was performed as previously described (Kuroiwa et al. 2020) with the antibodies listed in Table S3.

### 4.3 Retroviral Packaging and Infection

To establish MM231‐Venus, MM231‐TIE1, LM06‐shGFP, LM07‐shGFP, LM07‐TIE1KD#1, and LM07‐TIE1KD#2 cell lines via retroviral infection, Plat‐E cells were seeded into six‐well plates and transfected with the following plasmids: pMXs‐Venus‐IRES‐Puromycin^R^, pMXs‐TIE1‐IRES‐Puromycin^R^, pSuper‐shGFP‐Puromycin^R^, pSuper‐shTIE1#1‐Puromycin^R^, and pSuper‐shTIE1#2‐Puromycin^R^, using 4 μg of polyethylenimine (PEI; Polysciences Inc., PA, USA). Viral infection was carried out as previously described (Saito et al. 2012). To select for successfully transduced cells, 1 μg/mL puromycin (Fujifilm Wako) was added to the culture medium for 2 days. The target sequences of the shRNAs are listed in Table S4.

### 4.4 Animal Experiments and Bioluminescence Imaging

A total of 1.0 × 10^6^ cells were transplanted into the fourth mammary fat pads of 6‐week‐old NOD‐SCID female mice (Charles River Laboratories Japan Inc.), as previously described (Nakayama et al. 2017). Following injection, primary tumor growth and metastasis formation were monitored weekly using the IVIS system (IVIS Lumina XR3; PerkinElmer, Waltham, MA, USA). Mice were intraperitoneally injected with 200 μL of D‐Luciferin (15 mg/mL; Gold Biotechnology Inc.), and luminescence was recorded under anesthesia (2.5% isoflurane; Fujifilm Wako). Tumor volume was calculated using formula (1), where *x*, *y*, and *z* represent orthogonal tumor axes measured using a vernier micrometer.

(1) $$\text{Tumor volume}=\frac{4}{3}\pi \times \frac{x}{2}\times \frac{\;y}{2}\times \frac{z}{2}$$

At 7–8 weeks post‐injection, all mice were sacrificed, and their lungs were excised. The lungs were transferred to a 12‐well plate containing 900 μL of D‐PBS (−) (Fujifilm Wako) and 100 μL of D‐Luciferin (15 mg/mL), followed by ex vivo luminescence imaging using IVIS. All animal experiments were approved by the Animal Committee of Waseda University (WD22‐088, A22‐080, WD23‐084, A23‐074).

### 4.5 Histological Analysis

Excised tumors were fixed in 4% paraformaldehyde (PFA), paraffin embedded, and sectioned into 4 μm‐thick slices. The paraffin sections were deparaffinized in xylene (Fujifilm Wako) then rehydrated through a graded ethanol series (Fujifilm Wako) decreasing from 100% to 50%, followed by distilled water.

For IHC analysis, antigen retrieval was performed in 10 mM citrate buffer (pH 6.0) (Fujifilm Wako) containing 0.05% Tween 20 (Takara Bio Inc., Shiga, Japan) at 90°C–100°C for 30 min. Sections were then permeabilized with 0.1% Triton X‐100 in PBS (Fujifilm Wako). Endogenous peroxidase activity was blocked using 3% H_2_O_2_ (Fujifilm Wako) for 5 min. Following this, sections were incubated with 2.5% normal horse serum (Vector Laboratories) for 20 min at room temperature. Primary antibody against CC3 (9661, Cell Signaling Technology, 1:400) was applied, and sections were incubated overnight at 4°C. Subsequently, sections were incubated with secondary antibody solutions for 20 min at room temperature, followed by incubation with VECTASTAIN ABC Reagent (Vector Laboratories) for 1 h at room temperature. Staining was performed using the ImmPACT DAB EqV substrate kit (Vector Laboratories), followed by counterstaining with Mayer's hematoxylin (NACALAI TESQUE, INK.) for 5 min. Sections were then dehydrated through a series of graded ethanol solutions (50%–100%), followed by xylene. Finally, the sections were mounted using MGK‐S (Matsunami Glass Ind. Ltd.).

For hematoxylin and eosin (HE) staining, deparaffinized and rehydrated sections were stained with Mayer's hematoxylin for 5 min. After rinsing in distilled water, the sections were stained with 1% eosin solution (NACALAI TESQUE, INC.) for 3 min. They were then sequentially washed with 100% ethanol, 90% ethanol, and xylene. Finally, the sections were mounted using the same method as for the IHC specimens.

### 4.6 Clinical Database Analysis

Copy number alteration analysis was conducted using clinical datasets from TCGA and ICGC (ICGC/TCGA Pan‐cancer Analysis of Whole Genomes Consortium 2020). Genomic profiling and survival analysis, based on the Kaplan–Meier method, were performed using data from the METABRIC (Curtis et al. 2012; Pereira et al. 2016; Rueda et al. 2019). Copy number alteration results and OncoPrint visualizations were generated via cBioPortal (Cerami et al. 2012). Survival analysis was performed using the “survival” package in R version 4.3.2 (https://www.r‐project.org/).

### 4.7 Statistical Analysis

Welch's *t*‐test, one‐way ANOVA, and Chi‐squared test were conducted using GraphPad Prism 7 (GraphPad Software). Log‐rank tests for survival analysis were also carried out using R version 4.3.2 (https://www.r‐project.org/).

## Author Contributions

J.N. and K.S. conceived and designed the study. K.A. and T.M. performed the experiments. K.A. and J.N. analyzed the data. S.W. and K.S. interpreted the data. K.A. wrote the manuscript. J.N. and K.S. revised the manuscript. All authors read and approved the final manuscript.

## Ethics Statement

The authors have nothing to report.

## Conflicts of Interest

The authors declare no conflicts of interest.

## Supporting information


**Table S1:** List of the number of CC3‐positive cells in primary tumors from cell line derived by MM231.
**Table S2:** List of the number of CC3‐positive cells in primary tumors from cell line derived by LM07.
**Table S3:** List of antibody used in this study.
**Table S4:** List of shRNA sequence used in this research.

**Figure S1:** (a) Complete set of in vivo luminescence images of primary tumors in OX model using MM231‐Venus cell lines (corresponding to Figure 1c). (b) Complete set of in vivo luminescence images of primary tumors in OX model using LM06‐shGFP cell lines (corresponding to Figure 1c). (c) Complete set of in vivo luminescence images of primary tumors in OX model using LM07‐shGFP cell lines (corresponding to Figure 1c). (d) Representative ex vivo luminescence images of lungs (left panel) and quantification of lung metastases (right panel) from mice orthotopically transplanted with LM07‐shGFP, LM07‐TIE1KD#1, and LM07‐TIE1KD#2 cell lines (*n* = 3 for each group). One‐way ANOVA followed by Tukey's multiple comparison test. All data are presented as mean ± SEM. n.s., not significant.
**Figure S2:** (a) Schematic of the image analysis method used to count CC3‐positive cells. (b) Representative images of HE staining, hematoxylin signal particles, and cleaved caspase‐3 (CC3)‐positive cells in primary tumors of MM231‐Venus and MM231‐TIE1 groups (*n* = 4 each). (c) Representative images of HE staining, hematoxylin signal particles, and CC3‐positive cells in primary tumors of LM07‐shGFP, LM07‐TIE1KD#1, and LM07‐TIE1KD#2 cell lines (LM07‐shGFP: *n* = 4; LM07‐TIE1KD#1: *n* = 4; LM07‐TIE1KD#2: *n* = 4).

## Data Availability

The data that support the findings of this study are available on request from the corresponding author.
