# Supplementary material for: TIE1 Promotes Primary Tumor Growth by Inhibiting Apoptosis and Activating the AKT‐p70S6K Signaling Pathway in Breast Cancer
Source: Genes Cells. 2025 Nov 14;30(6):e70062. doi: 10.1111/gtc.70062 (PMC12617338; doi:10.1111/gtc.70062)
Supplement: Supplementary file 2 — Figure S1: (a) Complete set of in vivo luminescence images of primary tumors in OX model using MM231‐Venus cell lines (corresponding to Figure 1c). (b) Complete set of in vivo luminescence images of primary tumors in OX model using LM06‐shGFP cell lines (corresponding to Figure 1c). (c) Complete set of in vivo luminescence images of primary tumors in OX model using LM07‐shGFP cell lines (corresponding to Figure 1c). (d) Representative ex vivo luminescence images of lungs (left panel) and quantification of lung metastases (right panel) from mice orthotopically transplanted with LM07‐shGFP, LM07‐TIE1KD#1, and LM07‐TIE1KD#2 cell lines (n = 3 for each group). One‐way ANOVA followed by Tukey's multiple comparison test. All data are presented as mean ± SEM. n.s., not significant. Figure S2: (a) Schematic of the image analysis method used to count CC3‐positive cells. (b) Representative images of HE staining, hematoxylin signal particles, and cleaved caspase‐3 (CC3)‐positive cells in primary tumors of MM231‐Venus and MM231‐TIE1 groups (n = 4 each). (c) Representative images of HE staining, hematoxylin signal particles, and CC3‐positive cells in primary tumors of LM07‐shGFP, LM07‐TIE1KD#1, and LM07‐TIE1KD#2 cell lines (LM07‐shGFP: n = 4; LM07‐TIE1KD#1: n = 4; LM07‐TIE1KD#2: n = 4). [file GTC-30-0-s002.pptx]

## Slide 1
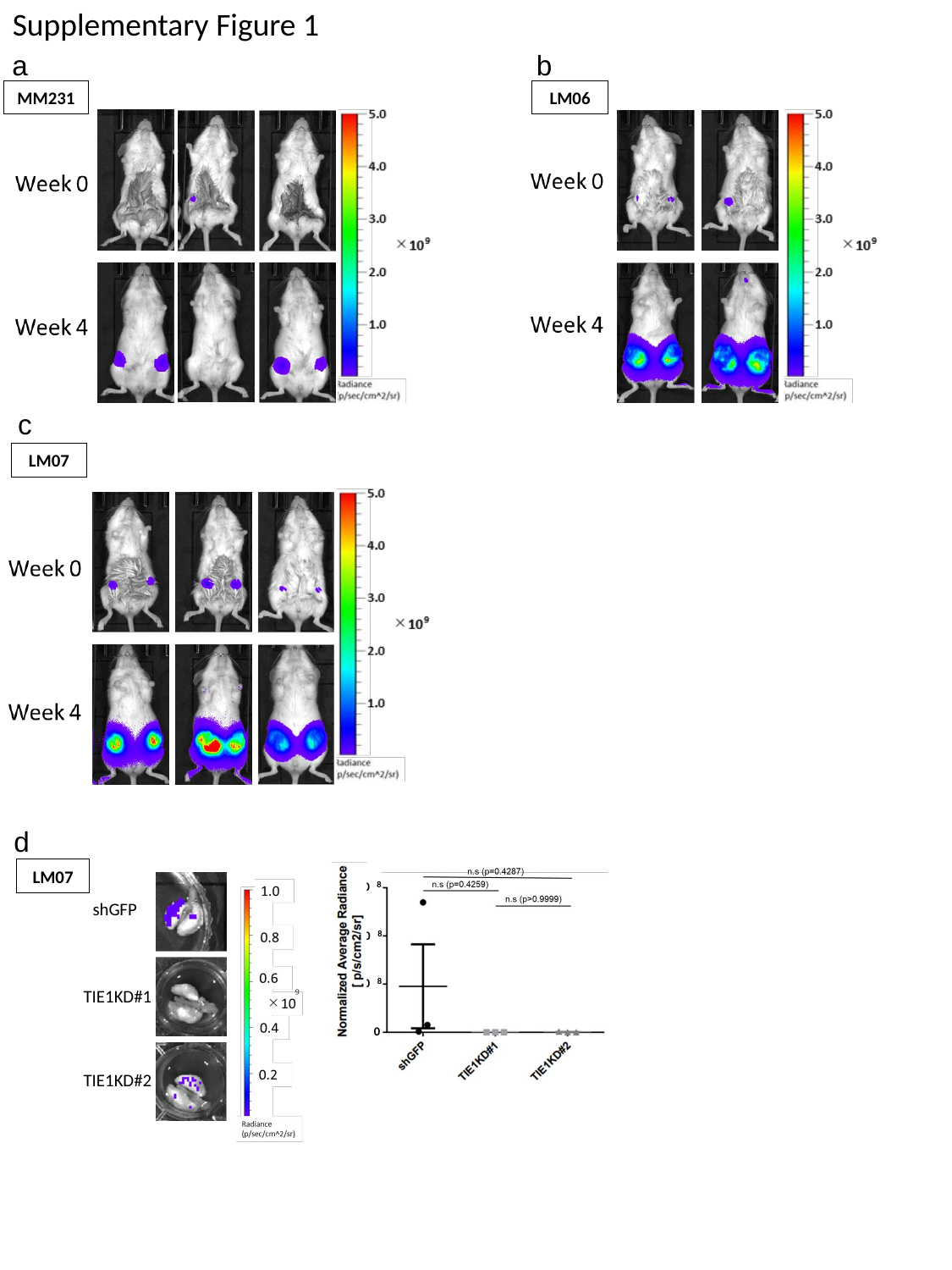

Supplementary Figure 1
a
b
MM231
LM06
c
LM07
d
LM07
6×10
8
shGFP
4×10
8
2×10
8
TIE1KD#1
0
TIE1KD#2

## Slide 2
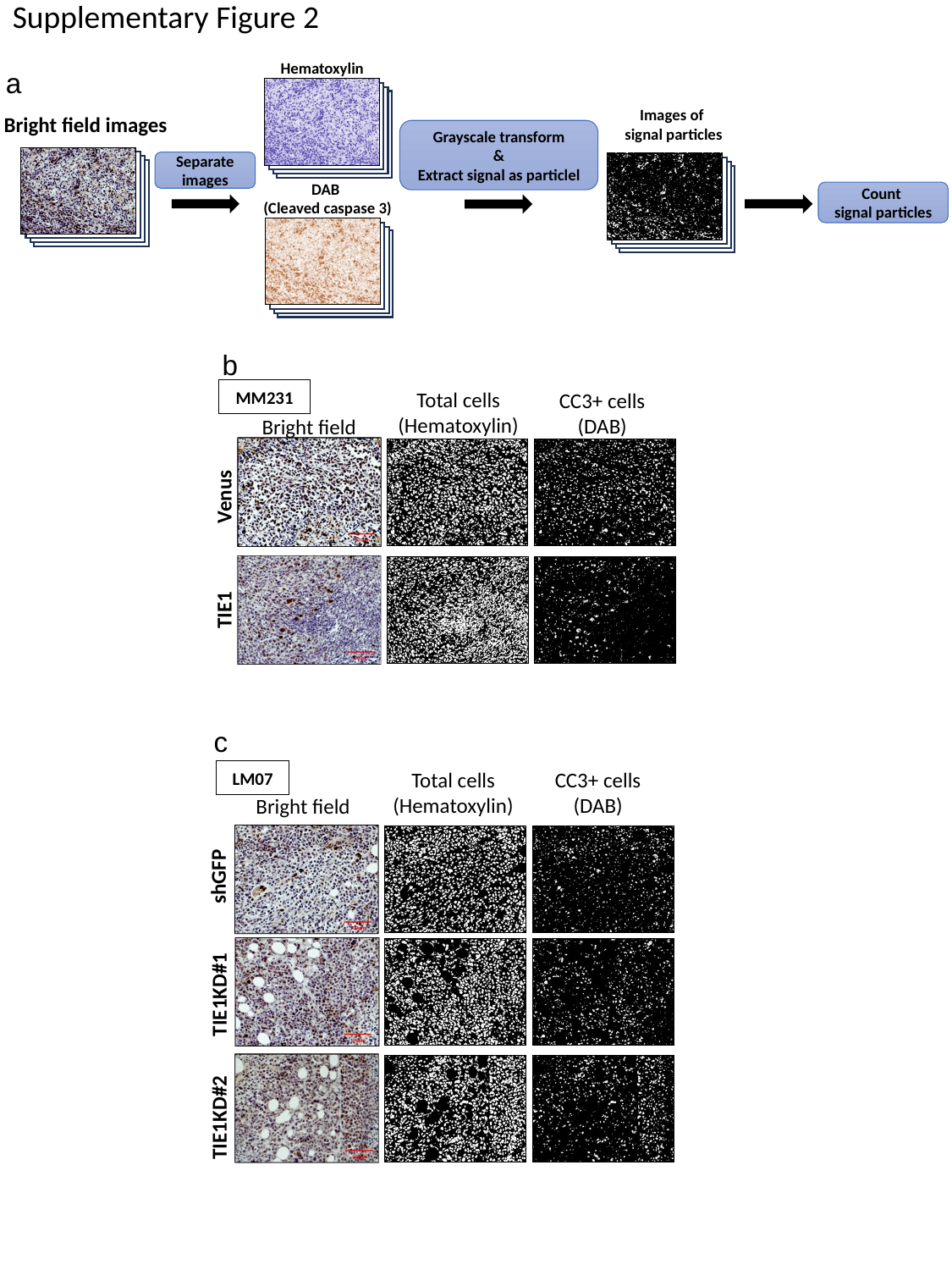

Supplementary Figure 2
Hematoxylin
a
Images of
signal particles
Bright field images
Grayscale transform
&
Extract signal as particlel
Separate images
DAB
(Cleaved caspase 3)
Count
signal particles
b
MM231
Total cells
(Hematoxylin)
CC3+ cells
(DAB)
Bright field
Venus
TIE1
LM05
c
Total cells
(Hematoxylin)
CC3+ cells
(DAB)
LM07
Bright field
shGFP
TIE1KD#1
TIE1KD#2
